# Nutritional Geometric Profiles of Insulin/IGF Expression in *Drosophila melanogaster*

**DOI:** 10.1371/journal.pone.0155628

**Published:** 2016-05-12

**Authors:** Stephanie Post, Marc Tatar

**Affiliations:** 1 Department of Molecular Biology, Cell Biology and Biochemistry, Providence, RI, Brown University, United States of America; 2 Department of Ecology and Evolutionary Biology, Providence, RI, Brown University, United States of America; Inha University, REPUBLIC OF KOREA

## Abstract

Insulin/IGF signaling (IIS) in *Drosophila melanogaster* is propagated by eight *Drosophila* insulin-like peptides (dilps) and is regulated by nutrition. To understand how dietary protein and sugar affect dilp expression, we followed the analytical concepts of the Nutritional Geometric Framework, feeding *Drosophila* adults media comprised of seven protein-to-carbohydrate ratios at four caloric concentrations. Transcript levels of all dilps and three IIS-regulated genes were measured. Each dilp presented a unique pattern upon a bivariate plot of sugar and protein. *Dilp2* expression was greatest upon diets with low protein-to-carbohydrate ratio regardless of total caloric value. *Dilp5* expression was highly expressed at approximately a 1:2 protein-to-carbohydrate ratio and its level increased with diet caloric content. Regression analysis revealed that protein-to-carbohydrate ratio and the interaction between this ratio and caloric content significantly affects dilp expression. The IIS-regulated transcripts *4eBP* and *InR* showed strikingly different responses to diet composition: *4eBP* was minimally expressed except when elevated at low caloric diets. *InR* expression increased with protein level, independent of caloric content. Values of published life history traits measured on similar diets revealed correlations between egg production and the expression of *dilp8 4eBP*, while low protein-to-carbohydrate ratio diets associated with long lifespan correlated with elevated *dilp2*. Analyzing how nutient composition associates with dilp expression and IIS reveals that nutritional status is modulated by different combinations of insulin-like peptides, and these features variously correlate to IIS-regulated life history traits.

## Introduction

Insulin/Insulin-like Growth Factor signaling (IIS) in the fruit fly *Drosophila melanogaster* is mediated by eight *Drosophila* insulin-like peptides (dilps) that signal through a common tyrosine kinase receptor INR (insulin/IGF receptor). *Dilps* and *InR* are homologous to insulin, insulin-like growth factor and their respective receptors in mammals [1]. Ligand-activated INR interacts with insulin receptor substrate IRS (*chico*, homolog of human IRS1-4) to initiate canonical PI3K and Akt signaling, and subsequently repress the forkhead transcription factor dFOXO [2]. The eight dilps are variously expressed across the life cycle, development and tissues [1,3]. Messenger RNA of *dilp1*, *dilp2*, *dilp3* and *dilp5* are predominantly expressed in median neurosecretory cells (MNCs; insulin producing cells, IPC) of the adult brain. *Dilp5* mRNA is also produced in adult ovarian follicles and renal tubules while *dilp3* is expressed in the midgut [1,4,5]. *Dilp6* mRNA is produced in adult and larval fat body, a tissue with adipose and liver-like functions [6,7]. *Dilp4* is expressed in embryo mesoderm [3]. *Dilp7* is expressed in the larval and adult central nervous system [3] and *dilp8* has been observed during pupal development [8,9].

Flies mutant for specific dilp loci have been used to explore their growth and metabolic functions, although interpreting outcomes is complicated by compensatory increase or decrease of various dilps when one locus is mutated. DILP2 peptide is inferred to modulate circulating carbohydrates because *dilp2* mutants have elevated hemolymph sugar [10]. A similar phenotype was reported from flies where the MNCs were ablated and rescue was subsequently achieved by exogenous expression of *dilp2* [11]. *Dilp2* has notably been associated with regulation of longevity. *Dilp2* mRNA and peptide are reduced in genetic manipulations that extend adult lifespan [7,12] and lifespan is extended in *dilp2* mutant adults [10]. Whether *dilp2* directly controls these phenotypes remains somewhat uncertain because mutation of *dilp2* simultaneously increases *dilp3* and *dilp5* expression [10,13]. Mutant flies that lack *dilp2*, *dilp3* and *dilp5* together forestall this compensatory expression: homozygote mutants no longer show extended lifespan, although heterozygote animals are slightly long-lived [10].

The functions of *dilp6* have been elucidated by analyzing mutants and with over-expression. *Dilp6* is critical for larval development and responds to the maturation hormone ecdysone [14]. *Dilp6* null mutants have slightly elevated lipid levels, suggesting that *dilp6* controls lipid storage and use [10]. In adults, overexpressing *dilp6* extends lifespan and increases fat and glycogen [7]. However, whether these effects are directly caused by *dilp6* is unknown because MSC production of *dilp2* and *dilp5* are reduced when *dilp6* is overexpressed in fat bodies [7].

Given the complex compensatory expression among dilps upon mutation, here we sought to understand how these peptides are expressed in the physiological context of wildtype animals fed different diets. In early reports, starvation reduced larval *dilp3* and *dilp5* but not *dilp2* [15]. With larvae and adults, *dilp5* but not *dilp2* was reduced when animals were maintained on yeast-restricted or all-component diluted diets [16–18]. On the other hand, starvation increased *dilp6* expression in larvae and adults while *dilp2* and *dilp5* were decreased or unchanged [6,7]. Together, these observations suggest that dilps uniquely mediate distinct metabolic roles: glucose metabolism by *dilp2*, lipid storage by *dilp6*, lipid metabolism by *dilp3*, and response to protein by *dilp5* [19].

To explore this perspective, we measured all dilp mRNAs in adults fed diets that varied by protein-to-carbohydrate ratio at four levels of caloric content. This design follows the analytical approach of the Geometric Nutritional Framework to separate the impact of nutrient composition from caloric content upon continuous traits [20–23]. In particular, we applied the dietary regimen of Lee *et al*. [24] where *Drosophila* adults were fed 28 diets of seven protein-to-carbohydrate ratios at four caloric concentrations. In that report, lifespan was maximized by a relatively low protein-to-carbohydrate (1:16) intake regardless of caloric intake, fecundity was maximized on a higher protein-to-carbohydrate ratio intake (1:2), and fitness was greatest at an intermediate protein-to-carbohydrate intake (1:4).

Although it is widely believed that caloric restriction modulates aging by reducing insulin/IGF signaling, evidence to support this theory is actually sparse [16,25,26]. *Foxo* mutants robustly extend lifespan when diet restricted [16]. Likewise, *dilp* mutants have little effect on the ability of DR to slow aging [10,17]. These counter-intuitive outcomes might be caused by the compensatory nature of dilp expression in mutant animals, and this further recommends that we study wildtype adults under different physiological conditions to test the hypothesis that insulin/IGF signaling is minimized upon diets where the ratio of protein to carbohydrate maximizes lifespan. Surprisingly, *dilp2* mRNA is most *elevated* upon diets with low protein-to-carbohydrate ratios, contrary to expectation if reduced *dilp2* is associated with longevity assurance. On the other hand, *dilp5* expression is reduced on diets with low protein-to-carbohydrate ratios, while *dilp8* is highly expressed on all diets except upon those with very low calorie level. Life history traits measured and reported for these diets reveal a positive correlation between egg production and expression of *dilp8*, a positive association between lifespan and expression of *dilp1* and *dilp2*, and a weak negative correlation between *dilp5* expression and lifespan.

## Materials and Methods

### Fly husbandry and nutritional geometry design

Outbred flies of the stock *yw*^R^ were maintained and reared at 25°C, 40% relative humidity and 12h light/dark cycle. Flies were reared on agar-based diet with cornmeal (5.2%), sucrose (11.0%), autolyzed yeast (2.5%; SAF brand) and agar (0.79%) (w/v in 100 mL water) with 0.2% Tegosept (methyl4-hydroxybenzoate, Sigma, St Louis, MO, USA) as an antifungal agent. After eclosion, flies were mated for two days after which females were separated and placed on a series of 28 diets (day 0) (Table 1). These diets used the ratios and energetic content as reported by Lee *et al*. [24], but here the nutrients were provided in solid agar-based media rather than liquid food. Yeast extract (MP Biomedical) and sucrose were combined with agar (0.79%) in the amounts detailed in Table 1. Ten females were put in each vial, three vials per diet. Flies were transferred to new vials at day 2 and day 4. The three biological replicates were pooled on day 5 and flies were homogenized in Trizol reagent using a TissueLyser (Qiagen). Gene expression from pooled biological samples is estimated to average the gene expression of the separate biological samples, according to the biological averaging assumption [27,28].

**Table 1:** Diet composition.

[Y+S]	Y:S^a^	g Yeast	g Sugar	P:C^b^	g Protein	g Carb	Diet #
*45 g/L*							
	0:1	0	9	0:1	0	9	1
	1:7	1.125	7.875	1:16	0.50625	8.145	2
	1:3.4	2.04	6.95	1:8	0.918	7.4396	3
	1:1.6	3.46	5.54	1:4	1.557	6.3704	4
	1:0.7	5.29	3.71	1:2	2.3805	4.9796	5
	1:0.2	7.5	1.5	1:1	3.375	3.3	6
	1:0	9	0	1.9:1	4.05	2.16	7
*90 g/L*							
	0:1	0	18	0:1	0	18	8
	1:7	2.25	15.75	1:16	1.0125	16.29	9
	1:3.4	4.08	13.9	1:8	1.836	14.8792	10
	1:1.6	6.92	11.08	1:4	3.114	12.7408	11
	1:0.7	10.58	7.42	1:2	4.761	9.9592	12
	1:0.2	15	3	1:1	6.75	6.6	13
	1:0	18	0	1.9:1	8.1	4.32	14
*180 g/L*							
	0:1	0	36	0:1	0	36	15
	1:7	4.5	31.5	1:16	2.025	32.58	16
	1:3.4	8.16	27.8	1:8	3.672	29.7584	17
	1:1.6	13.84	22.16	1:4	6.228	25.4816	18
	1:0.7	21.16	14.84	1:2	9.522	19.9184	19
	1:0.2	30	6	1:1	13.5	13.2	20
	1:0	36	0	1.9:1	16.2	8.64	21
*360 g/L*							
	0:1	0	72	0:1	0	72	22
	1:7	9	63	1:16	4.05	65.16	23
	1:3.4	16.32	55.6	1:8	7.344	59.5168	24
	1:1.6	27.68	44.32	1:4	12.456	50.9632	25
	1:0.7	42.32	29.68	1:2	19.044	39.8368	26
	1:0.2	60	12	1:1	27	26.4	27
	1:0	72	0	1.9:1	32.4	17.28	28

Diets are composed of four total caloric contents ([Y+S]) at seven protein-to-carbohydrate ratios.

^a^Y:S = yeast-to-sugar ratio

^b^P:C = protein-to-carbohydrate ratio

### Quantitative RT-PCR

Total RNA was extracted from whole flies (30 per sample) in Trizol (Invitrogen, Grand Island, NY, USA) and treated with Turbo DNase (Invitrogen). RNA was quantified with a NanoDrop ND-1000 (Thermo Fisher Scientific Inc., Wilmington, DE, USA) and reverse-transcribed with iScript cDNA synthesis (Bio-Rad Laboratories, Inc., Hercules, CA, USA). Quantitative RT-PCR was conducted with SYBR Green PCR master mix (Applied Biosystems, Carlsbad, CA, USA) and measured on an ABI prism 7300 Sequence Detection System (Applied Biosystems). mRNA abundance was calculated by comparative CT relative to ribosomal protein 49 (RP49). Although there are concerns about choice of reference gene in diet studies, RP49/RPL32 is reported as the most stable option [29], and we verified that RP49 is not sensitive to diet by viewing and analyzing the raw CT values (data not shown). We therefore chose RP49 as the reference in all relative gene expression analyses. Primer sequences are listed in S3 Table.

### Data Analysis

Expression data for each gene was normalized to its value on diet #7. Normalized values were visualized on landscapes using nonparametric thin plate splines in R using the “fields” package as performed previously [30]. For quantitative analysis, gene expression was treated as response variables in multivariate multiple regression conducted in R using *lm* and *Manova* functions, evaluating linear models (protein-to-carbohydrate ratio or caloric content) and nonlinear models (P:C ratio-by-calorie interaction, and ratio-by-ratio and calorie-by-calorie quadratic functions). Coefficients were compared among dilps by ANOVA (http://statpages.org/anova1sm.html). Post hoc pairwise tests were conducted by the Holm-Sidak student’s *t*-test using mean and standard error (R *tsum*.*test* function, “BSDA” package). Data for lifespan and egg production reported by Lee *et al*. [24] for females upon the same matrix of diets were compared to our current measures of gene expression by Pearson’s product moment correlation coefficient, adjusting *t* for sample size by t = (r-ρ)/√[(1-r^2^)/(n-2) to calculate significance with a two-tailed students *t*-test.

## Results

**Fig 1:**
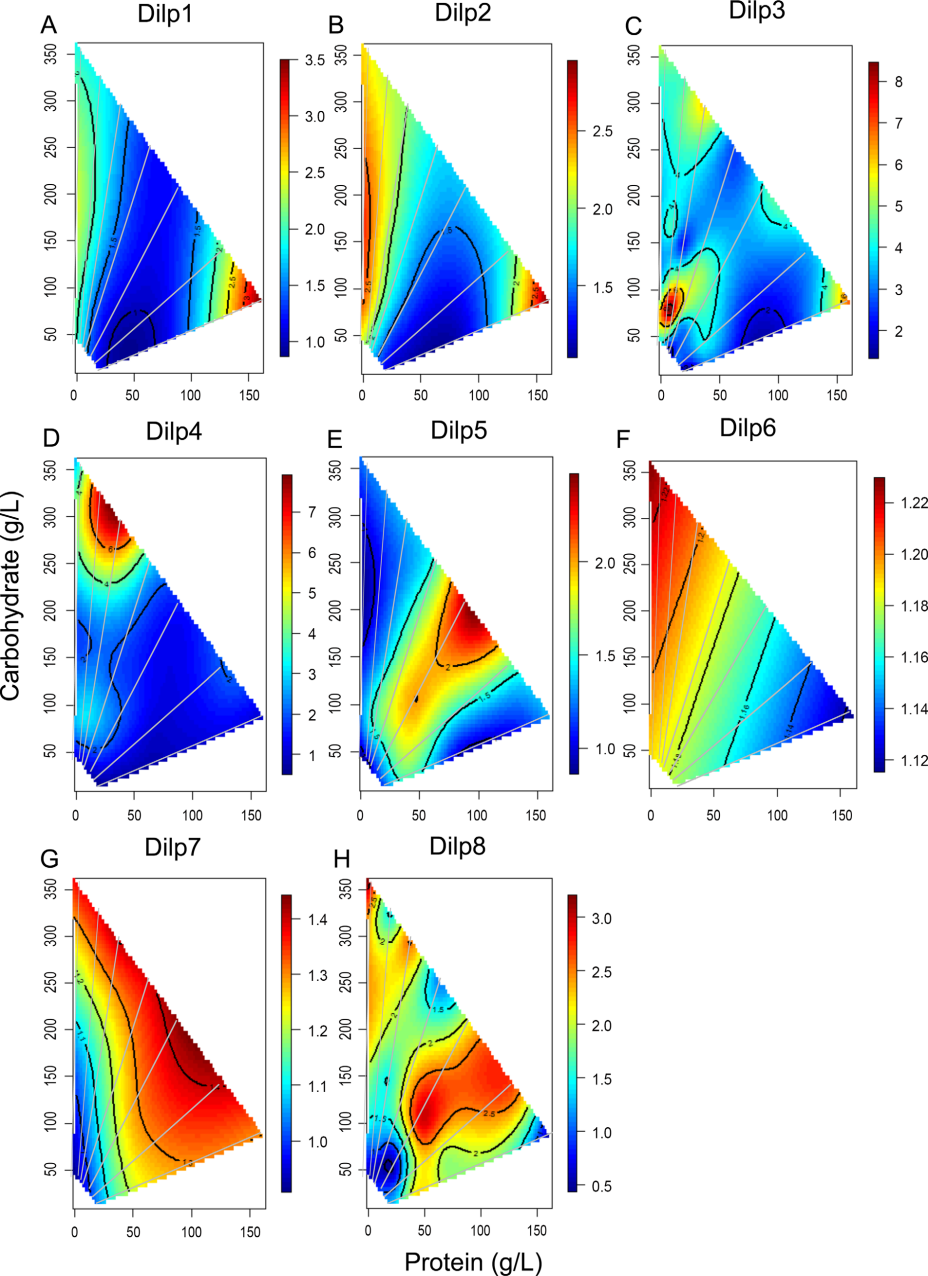
Dilp gene expression across the nutritional geometric framework surface. Gene expression was plotted against protein and carbohydrate content of 28 diets by nonparametric thin plate splines in R. Gray lines represent the seven protein-to-carbohydrate ratios. Heat maps from blue to red represent the normalized level of gene expression from lowest to highest. A) *Dilp1*, B) *Dilp2*, C) *Dilp3*, D) *Dilp4*, E) *Dilp5*, F) *Dilp6*, G) *Dilp7* and H) *Dilp8*.

Each dilp produces a distinct expression pattern across the diet landscape (Fig 1). Several cases show maximum expression along specific vectors of dietary protein-to-carbohydrate ratio, where the highest ridge of expression aligns with a particular ratio, denoted by gray lines. Notably, *dilp2* mRNA is greatest at low protein diets where the protein-to-carbohydrate ratio is approximately 1:16 (Fig 1B). While the absolute levels of *dilp1* mRNA are lower than *dilp2* (S1 Table and S1 Fig), these two dilps show similar patterns across diets (Fig 1A, Table 2). In contrast, *dilp5* (Fig 1E) is highly expressed at a protein-to-carbohydrate ratio of approximately 1:2, and upon diets of high caloric value, creating a rising ridge contour. *Dilp3* (Fig 1C) is maximized on diets with low caloric content at a protein-to-carbohydrate ratio of approximately 1:8, and its pattern is moderately correlated to *dilp2* expression (Table 2). *Dilp6* mRNA (Fig 1F) is maximized at low protein, high calorie diets and generally decreases with increasing protein-to-carbohydrate ratio.

**Table 2:** Correlation coefficients among gene expression and life history traits.

		Egg	Dilp1	Dilp2	Dilp3	Dilp4	Dilp5	Dilp6	Dilp7	Dilp8	Upd2	4eBP	InR
**Lifespan**	R^2^	0.473	0.133	0.359	0.378	0.616	0.038	0.040	0.056	0.276	-0.153	-0.404	-0.382
	*p value*	*0*.*011*	*0*.*499*	*0*.*061*	*0*.*047*	*0*.*000*	*0*.*846*	*0*.*841*	*0*.*779*	*0*.*155*	*0*.*437*	*0*.*033*	*0*.*045*
**Egg**	R^2^		-0.135	-0.045	0.119	0.349	0.363	-0.059	0.299	0.330	-0.109	-0.432	-0.180
	*p value*		*0*.*492*	*0*.*821*	*0*.*545*	*0*.*069*	*0*.*058*	*0*.*765*	*0*.*122*	*0*.*087*	*0*.*581*	*0*.*022*	*0*.*359*
**Dilp1**	R^2^			0.820	0.370	0.085	-0.198	0.343	-0.321	-0.173	0.683	-0.235	0.487
	*p value*			*0*.*000*	*0*.*053*	*0*.*669*	*0*.*312*	*0*.*074*	*0*.*096*	*0*.*377*	*0*.*000*	*0*.*229*	*0*.*009*
**Dilp2**	R^2^				0.723	0.191	0.087	0.439	-0.173	-0.081	0.544	-0.240	0.220
	*p value*				*0*.*000*	*0*.*329*	*0*.*660*	*0*.*020*	*0*.*377*	*0*.*684*	*0*.*003*	*0*.*218*	*0*.*261*
**Dilp3**	R^2^					0.404	0.523	0.389	0.266	0.121	0.356	-0.143	0.011
	*p value*					*0*.*033*	*0*.*004*	*0*.*041*	*0*.*171*	*0*.*540*	*0*.*063*	*0*.*467*	*0*.*958*
**Dilp4**	R^2^						0.101	0.157	0.168	0.066	-0.141	-0.134	-0.215
	*p value*						*0*.*611*	*0*.*424*	*0*.*392*	*0*.*737*	*0*.*475*	*0*.*496*	*0*.*272*
**Dilp5**	R^2^							0.399	0.602	0.379	-0.030	-0.200	-0.011
	*p value*							*0*.*035*	*0*.*001*	*0*.*047*	*0*.*880*	*0*.*307*	*0*.*957*
**Dilp6**	R^2^								0.036	0.016	-0.064	-0.147	-0.073
	*p value*								*0*.*855*	*0*.*934*	*0*.*747*	*0*.*456*	*0*.*714*
**Dilp7**	R^2^									0.785	-0.248	-0.232	-0.141
	*p value*									*0*.*000*	*0*.*204*	*0*.*235*	*0*.*473*
**Dilp8**	R^2^										-0.299	-0.558	-0.230
	*p value*										*0*.*122*	*0*.*002*	*0*.*240*
**Upd2**	R^2^											-0.147	0.725
	*p value*											*0*.*456*	*0*.*000*
**4eBP**	R^2^												0.037
	*p value*												*0*.*851*

For p values reported as 0.000, p<0.0005

The expression patterns of other dilps are maximized or minimized in small regions of the dietary space. *Dilp4* is greatest on high sugar, low protein and high caloric diets and uniformly expressed at low levels on other diets (Fig 1D). *Dilp7* is expressed on most diets, except when it is reduced on very low calorie food (Fig 1G). The absolute level of *dilp8* transcription was much higher than any other dilp gene (S1 Table) but showed a bifurcated pattern of expression similar to *dilp4* when protein levels are low, and similar to *dilp5* when protein levels are high (Fig 1H); overall, *dilp8* is most correlated to *dilp7* expression (Table 2).

Multivariate multiple regression analysis was used to evaluate the overall effect of individual diet variables (P:C ratio, caloric content), and their interaction (P:C ratio-by-caloric content) upon net, collective dilp expression (Table 3). Dilps and related insulin/IGF signaling factors (*4eBP*, *InR* and *Upd2*) were significantly affected by protein-to-carbohydrate ratio (linear and quadratic models) and by the P:C ratio-by-caloric content interaction (S2 Table; p = 0.026, p = 0.012 and p = 0.011 respectively). Caloric content was significantly associated with the overall expression of dilps in a quadratic model (S2 Table; p = 0.043). Considering the expression of each gene individually, protein-to-carbohydrate ratio linearly associated with *dilp1*, *dilp2*, *dilp3* and *Upd2*, while caloric content affected *dilp4*, *dilp7*, *dilp8*, *Upd2* and *4eBP* (Table 3, p<0.05). Caloric content significantly affected expression of *dilp5*, *dilp8* and *4eBP* when fitted to a quadratic model (C×C, Table 3, p<0.05). *Dilp1*, *dilp2*, *dilp8* and *Upd2* were significantly affected by the P:C ratio-by-caloric content interaction (Table 3, p<0.05).

**Table 3:** Estimated model effects of protein-to-carbohydrate ratio and caloric content on insulin/IGF signaling gene expression.

Response Variable	Linear effects		Nonlinear effects		
	P:C Ratio (R)	Caloric content (C)	R×R	C×C	R×C
***dilp1***					
Slope ± SE	-1.29 ± 0.38	-0.002 ± 0.0016	-0.20 ± 0.27	0.0015 ± 0.0014	0.0066 ± 0.0019
*t*	-3.35	-1.28	-0.74	1.10	3.48
P	0.003	0.21	0.47	0.28	0.002
***dilp2***					
Slope ± SE	-1.11 ± 0.30	-0.001 ± 0.001	-0.39 ± 0.20	0.0013 ± 0.0011	0.0043 ± 0.0015
*T*	-3.7	-0.85	-1.95	1.17	2.94
P	0.001	0.40	0.06	0.25	0.007
***dilp3***					
Slope ± SE	-2.10 ± 0.84	0.0004 ± 0.003	-0.82 ± 0.53	0.0046 ± 0.0028	0.0076 ± 0.004
*t*	-2.50	0.118	-1.55	1.64	1.87
P	0.02	0.91	0.13	0.11	0.07
***dilp4***					
Slope ± SE	-0.05 ± 1.17	0.01 ± 0.005	-1.15 ± 0.73	0.0077 ± 0.0037	-0.0065 ± 0.0056
*t*	-0.04	2.38	-1.57	2.06	-1.16
P	0.97	0.03	0.13	0.05	0.26
***dilp5***					
Slope ± SE	0.14 ± 0.31	0.0014 ± 0.0013	0.048 ± 0.18	0.0011 ± 0.00092	-0.00052 ± 0.0015
*t*	0.44	1.13	0.27	1.23	-0.35
P	0.67	0.27	0.79	0.023	0.73
***dilp6***					
Slope ± SE	-0.24 ± 0.20	-0.0004 ± 0.0008	-0.14 ± 0.11	-0.00002 ± 0.0006	0.00062 ± 0.0009
*t*	-1.22	-0.45	-1.23	-0.03	0.65
P	0.23	0.66	0.23	0.98	0.52
***dilp7***					
Slope ± SE	0.53 ± 0.26	0.0025 ± 0.001	0.16 ± 0.16	0.0013 ± 0.00083	-0.0022 ± 0.0012
*t*	2.06	2.37	1.0	1.55	-1.77
P	0.05	0.03	0.33	0.13	0.09
***dilp8***					
Slope ± SE	0.73 ± 0.39	0.0056 ± 0.0016	0.026 ± 0.27	0.0033 ± 0.0013	-0.0042 ± 0.0019
*t*	1.89	3.546	0.10	2.60	-2.24
P	0.07	0.0016	0.92	0.02	0.03
***Upd2***					
Slope ± SE	-9.29 ± 2.80	-0.025 ± 0.011	3.87 ± 2.51	0.018 ± 0.013	0.078 ± 0.013
*t*	-3.32	-2.22	1.54	1.32	5.78
P	0.003	0.04	0.14	0.20	0.000006
***4eBP***					
Slope ± SE	-0.10 ± 0.25	-0.0030 ± 0.0010	-0.067 ± 0.17	-0.0029 ± 0.00073	0.00022 ± 0.0012
*t*	-0.42	-3.0	-0.39	-3.93	0.18
P	0.68	0.006	0.70	0.0006	0.86
***InR***					
Slope ± SE	0.05 ± 0.11	-0.0004 ± 0.00043	0.20 ± 0.06	0.0001 ± 0.0004	0.0009 ± 0.00051
*t*	0.439	-0.94	3.17	0.24	1.78
P	0.66	0.34	0.004	0.81	0.09

**Fig 2:**
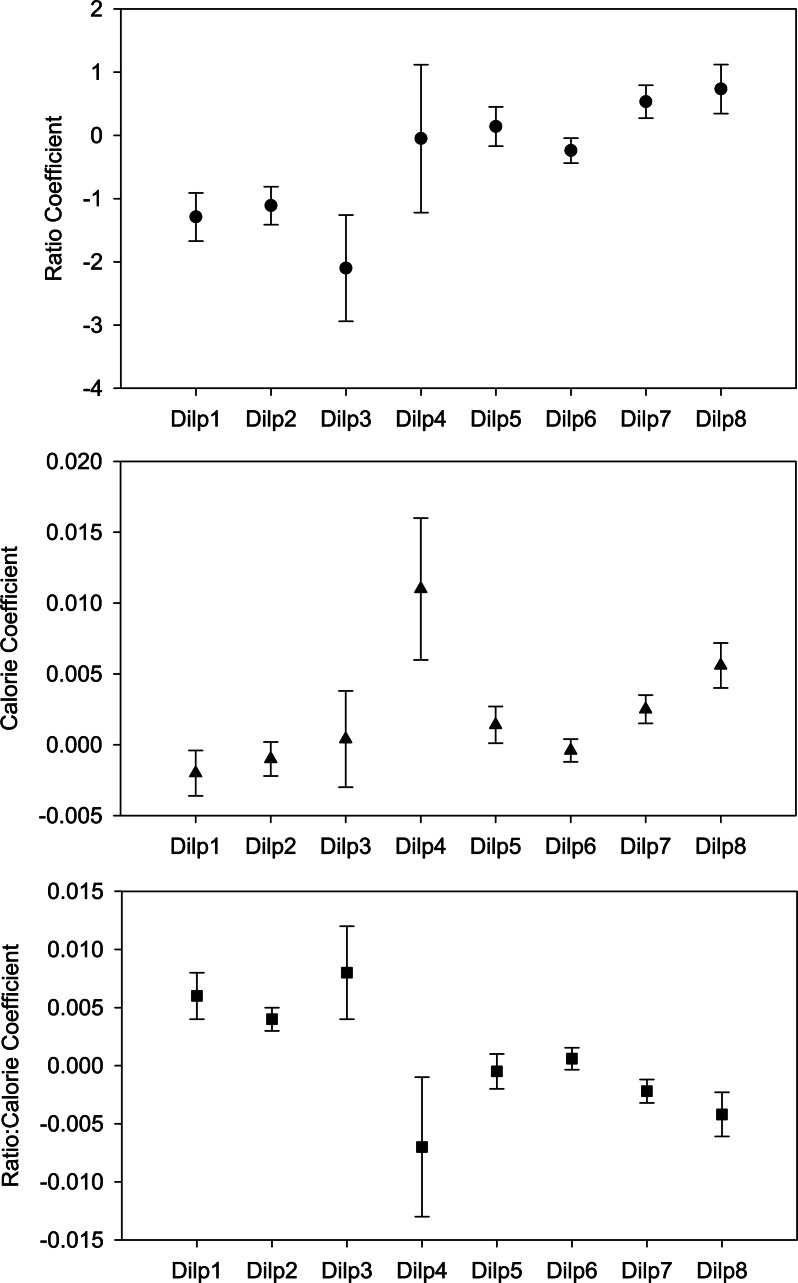
Protein-to-carbohydrate ratio, caloric content and P:C ratio-by-caloric content interaction affect dilp gene expression. The multivariate multiple regression functions’ coefficients were compared between dilps by ANOVA. Protein-to-carbohydrate ratio (top panel): p = 0.006 among dilps; significant differences upon pairwise post hoc test for *dilp3*-*dilp7* and *dilp3*-*dilp8*. Caloric content (middle panel): p = 0.004 among dilps; significant differences upon pairwise post hoc test for *dilp1*-*dilp4*, *dilp2*-*dilp4* and *dilp4*-*dilp6*. P:C ratio-by-caloric content interaction (bottom panel): p = 0.003 among dilps; significant difference upon pairwise post hoc test for *dilp1*-*dilp4* and *dilp3*-*dilp4*.

Among dilps, estimated parameters for protein-to-carbohydrate ratio, caloric content, and the interaction between P:C ratio and caloric content varied significantly (Fig 2). High protein-to-carbohydrate ratio (top panel) reduces *dilp1*, *dilp2* and *dilp3* expression, but increases expression of *dilp5*, *dilp6*, *dilp7* and *dilp8*. The P:C ratio-by-caloric content interaction (bottom panel) has similar clusters: *dilp1*, *dilp2* and *dilp3* have a positive coefficient, and *dilp5*, *dilp6*, *dilp7* and *dilp8* have negative or negligible coefficients.

**Fig 3:**
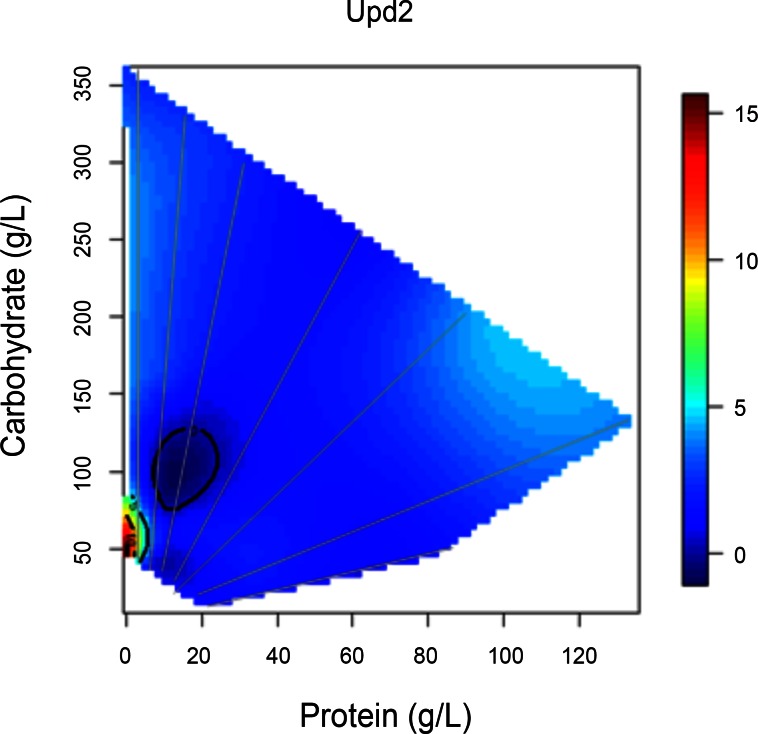
*Upd2* expression is increased on low protein:carbohydrate ratio diets. Gene expression was plotted against the protein and carbohydrate content of 28 diets by nonparametric thin plate splines in R. Gray lines represent the seven protein-to-carbohydrate ratios. Heat maps from blue to red represent the normalized level of gene expression from lowest to highest.

Several genes associated with *Drosophila* IIS show distinct expression patterns with respect to diet. *Unpaired-2* (*Upd2*) is a cytokine-like signaling molecule produced in *Drosophila* fat body [31] and midgut [32]. Fat body *Upd2* was reported to increase on high sugar diets and to regulate the release of DILP hormone from the brain [31]. Here, *Upd2* expression was elevated ~5-fold on low protein-to-carbohydrate diets and especially when caloric content was minimal (Fig 3; Excluding diet #28 as an outlier, S2 Fig, Grubbs/ESD test p<0.05). The *Upd2* surface pattern correlates moderately with that of *dilp1* and *dilp2* (Table 2).

**Fig 4:**
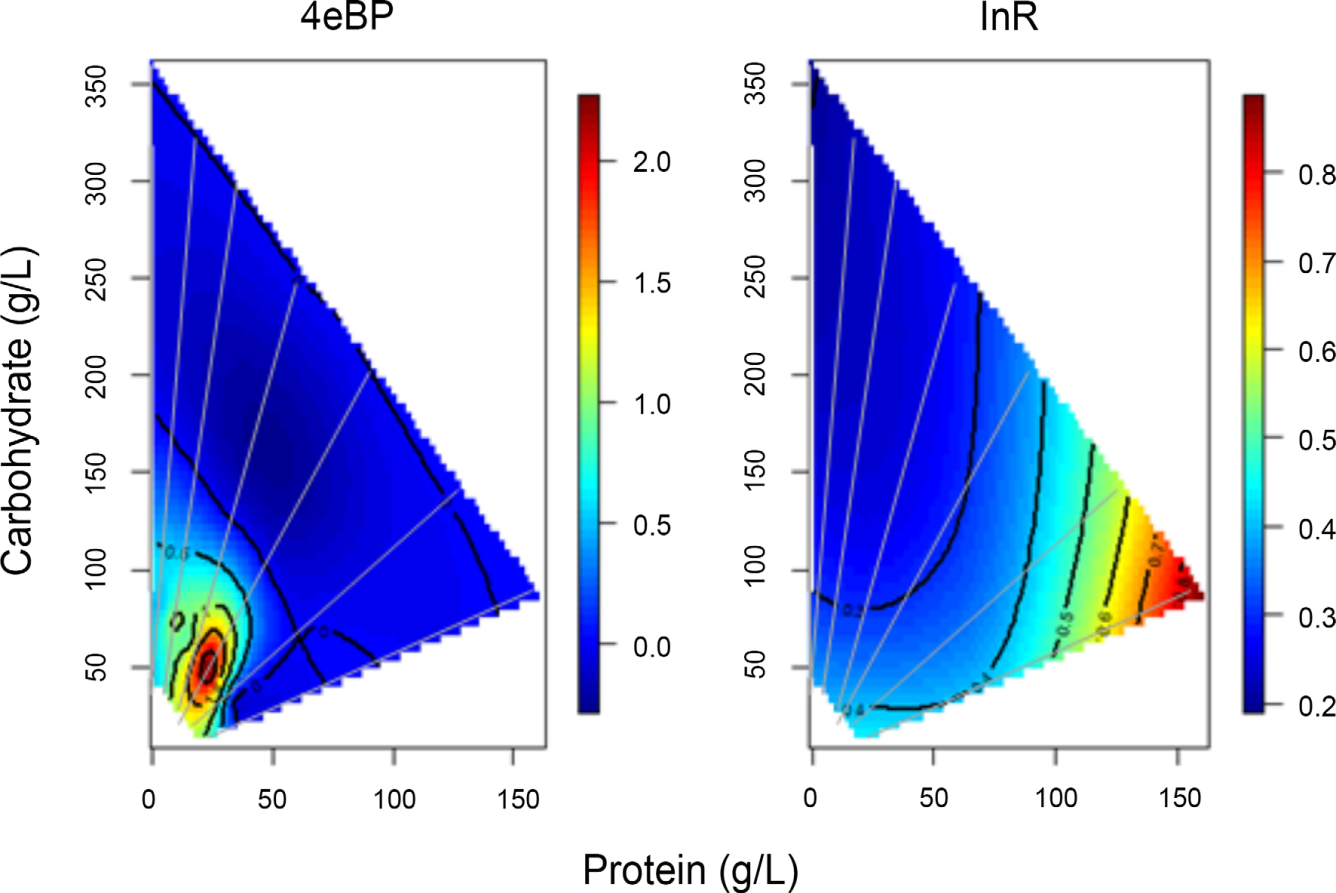
dFOXO transcriptional targets respond differently to diet composition. Gene expression was plotted against the protein and carbohydrate content of 28 diets by nonparametric thin plate splines in R. Gray lines represent the seven protein-to-carbohydrate ratios. Heat maps from blue to red represent the normalized level of gene expression from lowest to highest.

DILP1-7 are proposed to signal through a common insulin/IGF receptor (INR), and thus repress the transcription factor dFOXO. *4eBP* and *InR* are verified FOXO transcriptional targets [33,34], although *4eBP* is also strongly regulated by the TORC1 related transcription factor REPTOR [35]. *InR* and *4eBP* produce strikingly different expression surfaces on the dietary landscape (Fig 4). *4eBP* mRNA is greatest on diets with low caloric content and when the protein-to-carbohydrate ratio is approximately 1:2. In contrast, *InR* mRNA increases with dietary protein, independent of caloric content. *4eBP* expression was strongly yet inversely correlated with *dilp8* mRNA (Table 2), while *InR* mRNA was moderately correlated with *dilp1* (Table 2).

## Discussion

Eight dilps are produced and are measurable in adult female *Drosophila*. To date, the expression of these dilps in response to nutrition has been quantified using diets that differ by a single component (yeast) or by diluting all components. In these studies, *dilp5* expression was regulated by protein level while *dilp2* and *dilp3* mRNA were not affected by nutrients [16,17]. In contrast to single dimensional designs, Geometric Nutritional Framework analysis varies the proportion of nutritional components across defined ranges of caloric content. It provides a multidimensional approach to analyze how diet composition and quantity affect complex phenotypes such as longevity and gene expression. Geometric Framework analyses in *Drosophila* [24,36] as well as mammals [37] demonstrate that metabolism and longevity are modulated by particular ratios of protein to carbohydrate in the diet rather than by caloric content. The mechanism by which the ratio of protein to carbohydrate affects these traits is unknown, but is widely thought to involve the expression of insulin-like peptides.

We find with *Drosophila* that each *dilp* produces a distinctive expression surface relative to dietary protein-to-carbohydrate ratio and caloric content. The topography for *dilp2* and *dilp5* produce ridges of maximal expression at distinct protein-to-carbohydrate ratios of about 1:16 and 1:2 respectively. This pattern for *dilp2* may be consistent with previous reports where *dilp2* mRNA levels did not change in response to dietary yeast or total calories [16,17], because those studies used diets where *dilp2* is fairly constant. In contrast, *dilp5* is greatest on diets with a higher proportion of protein, and somewhat more so on diets with higher caloric content. This pattern may be consistent with studies that measured *dilp5* expression when dietary yeast (protein) was increased from low to moderate levels [16,17]. *Dilp2* and *dilp5* thus respond to nutrients in qualitatively different ways.

**Fig 5:**
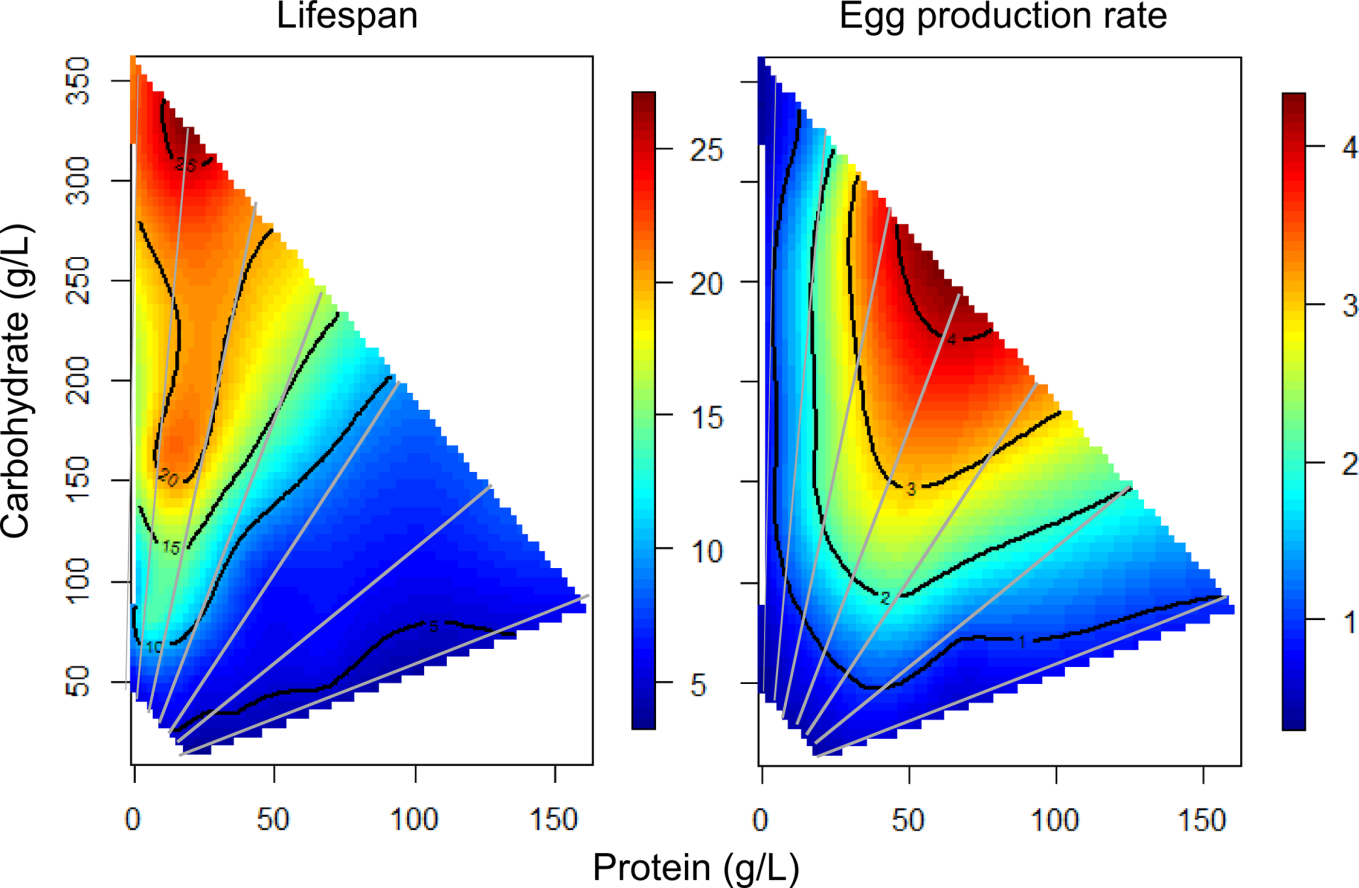
Reported measures of lifespan and fecundity associate with expression of specific dilps. Lifespan and fecundity data from Lee *et al*. [24] are plotted relative to diet nutrient content of 28 diets (used in Lee *et al*. and in the current study) by nonparametric thin plate splines in R. Gray lines represent seven protein-to-carbohydrate ratios. Heat maps from blue to red represent the phenotype value from lowest to highest.

These nutrient responses can be related to life history traits measured by Lee *et al*. [24] upon the same matrix of diets we employ in our current study. Although our wildtype strain (yw^R^) differs from that of Lee (Canton-S), meta-analysis of various *Drosophila* wildtype strains from different labs utilizing both liquid- and agar-based food delivery produced similar life history trait patterns mapped upon the Geometric Framework [24]. In Fig 5 we plot the egg production and lifespan data of Lee *et al*. [24] against the nutrient content of our common diets, noting that Lee originally plotted these traits relative to *nutrient intake* rather than to *diet content*. Based on diet content, longevity is maximized along the 1:16 protein-to-carbohydrate rail, as also seen when Lee *et al*. plotted this trait relative to nutrient intake. Here we now find that longevity and *dilp2* expression are correlated in a modest but positive fashion (Table 2, R^2^ = 0.36).

One potential way to understand this unexpected association considers how *Drosophila* use different food sources as adults. *Drosophila* need yeast-rich rotting fruit and vegetation to support egg production and larval development [38]. Such food sources are patchy in nature, and adults may fuel while searching by feeding upon common carbohydrate biased foods such as nectar [38]. In environments where protein is scarce, elevated *dilp2* will occur in a nutrient landscape while adult Drosophila are searching for protein-rich sites, and may thus induce physiology appropriate for longevity assurance while foraging in a patchy environment.

We are aware that the observed positive correlation between longevity and *dilp2* contrasts with studies where reduced *dilp2* is associated with longevity [10,12,39]. This difference is not readily explained but we note that mutation of any one specific dilp changes the expression of other dilps. Compensatory expression among these genes could affect metabolism and lifespan and confound how we interpret their individual function.

In contrast to *dilp2*, *dilp5* expression is greatest on diets where longevity is minimized and there is no correlation between the phenotypes). This is consistent with studies where reduced dietary yeast extend lifespan and simultaneously reduce *dilp5* expression [17]. While current models point to *dilp2* as most responsible for the control of aging, our observation suggests that *dilp5* accelerates aging under normal physiological conditions.

The expression patterns of *dilp1* and *dilp2* are strongly correlate (Table 2), suggesting that they may share physiological functions. No functions in the adult fly have yet been attributed to *dilp1*, possibly because of redundancy with highly expressed *dilp2*. Both *dilp1* and *dilp2* correlate moderately with expression of *Upd2*. *Upd2* is thought to non-cell autonomously regulate *dilp2* in IPCs in response to nutrition [31]; by extension, *Upd2* might also regulate *dilp1*. *Dilp3* expression is greatest at a simple maximum at approximately 1:8 protein-to-carbohydrate in low calorie diets. Thus, the four dilps produced in brain MSC produce three distinct expression patterns with respect to dietary protein and carbohydrate composition.

Other adult dilps are produced outside of the brain. Expression of *dilp6* is greatest on low protein-to-carbohydrate, high calorie diets. Previously, Skorupa *et al*. [36] observed increased triglyceride levels and adipose tissue volume with low protein-to-carbohydrate ratio and high sugar diets. High adiposity may elevate *dilp6* expression because *dilp6* is produced in fat body [6,7].

Although expressed in adults, *dilp4*, *dilp7* and *dilp8* are proposed to function predominantly in development [1,8,40,41]. However, because these dilp transcripts may occur as maternally deposited mRNAs in embryos within adult females, our interpretations of their adult function are tentative. *Dilp4* and *dilp7* are most abundant at extreme nutritional states, such as diets with very high protein-to-carbohydrate ratio or very high caloric content. These results suggest that *dilp4* and *dilp7* are not broadly sensitive to nutritional status.

In pupae, *dilp8* delays metamorphosis through control of ecdysone during adverse conditions such as injured discs [9]. Here, adult *dilp8* mRNA was surprisingly abundant on many diets (S1 Table). Notably, *dilp8* expression was negatively correlated with *4eBP* mRNA, which may reflect an inverse association with IIS signaling, or alternatively with *4eBP* induction by REPTOR that occurs when TOR is repressed [35]. Expression of *dilp8* was weakly correlated with egg production (Table 2). *Dilp8* in adult ovaries may regulate reproduction, although caloric content itself may coordinately reduce both traits.

Longevity in *Drosophila* is readily manipulated by ablating insulin-producing cells and through mutations of the signaling pathway [10,42–44]. Dietary restriction extends *Drosophila* lifespan [23], and Lee *et al*. [24] implicated protein-to-carbohydrate ratio as the specific, operative nutrient factor. Given these many observations, whether and how insulin-like peptides mediate the impact of diet upon longevity remains surprisingly unclear. Here we find that *dilp1* and *dilp2* are highly expressed at 1:16 protein-to-carbohydrate ratio, which presumably will inactive the pro-longevity FOXO transcription factor [12,45]. This ridge of expression correlates to elevated longevity in Lee *et al*. [24]. On the other hand, Skorupa *et al*. [36] demonstrated low caloric diets with a protein-to-carbohydrate close to 1:1 as most favorable for longevity. Such diets would occur in the lower left corner of our plots, where all dilp mRNA is reduced and *4eBP* expression is greatest. More recently, the longevity response to P:C ratio was measured using a holidic diet [46]: survival was optimized at protein-to-carbohydrate ratios of approximately 1:2 or 1:4. These ratios correspond to diets we find to have low overall dilp expression, in which case reduced net expression of all dilps may be required for longevity assurance.

An emerging theme among geometric framework analyses of lifespan emphasizes the importance of protein-to-carbohydrate ratio rather than caloric content as the operative nutrient feature [24,36,37,46]. Our analysis suggests that the interaction between protein-to-carbohydrate ratio and caloric content affects insulin/IGF signaling, and the corresponding life history traits. The geometric framework analysis permits finer scale dissection of how dilps are regulated and correlated with respect to diet and each other, and how these patterns may be disrupted when a single dilp is mutated. Ultimately, studies combining mutant and Geometric Framework approaches [20] are needed to provide a thorough analysis of individual dilp functions in life history traits and metabolism.

## Supporting Information

S1 Fig Feeding on normal food, wildtype flies express ~100-fold more dilp2 than dilp1 mRNA. (DOCX)

S2 Fig Upd2 expression along the geometric topography is hidden by extremely high expression at one outlier diet (Grubbs/ESD test p<0.05). (DOCX)

S1 Table Relative gene expression values are the average of three technical replicates and are standardized to RP49 expression. (PDF)

S2 Table Multivariate multiple regression MANOVA table. (DOCX)

S3 Table q-RT-PCR primer sequences. (DOCX)
