# Supplementary figures and images for: Nutritional Geometric Profiles of Insulin/IGF Expression in Drosophila melanogaster
Source: PLoS One. 2016 May 12;11(5):e0155628. doi: 10.1371/journal.pone.0155628 (PMC4865203; doi:10.1371/journal.pone.0155628)

S1 Fig. Feeding on normal food, wildtype flies express ~100-fold more dilp2 than dilp1 mRNA


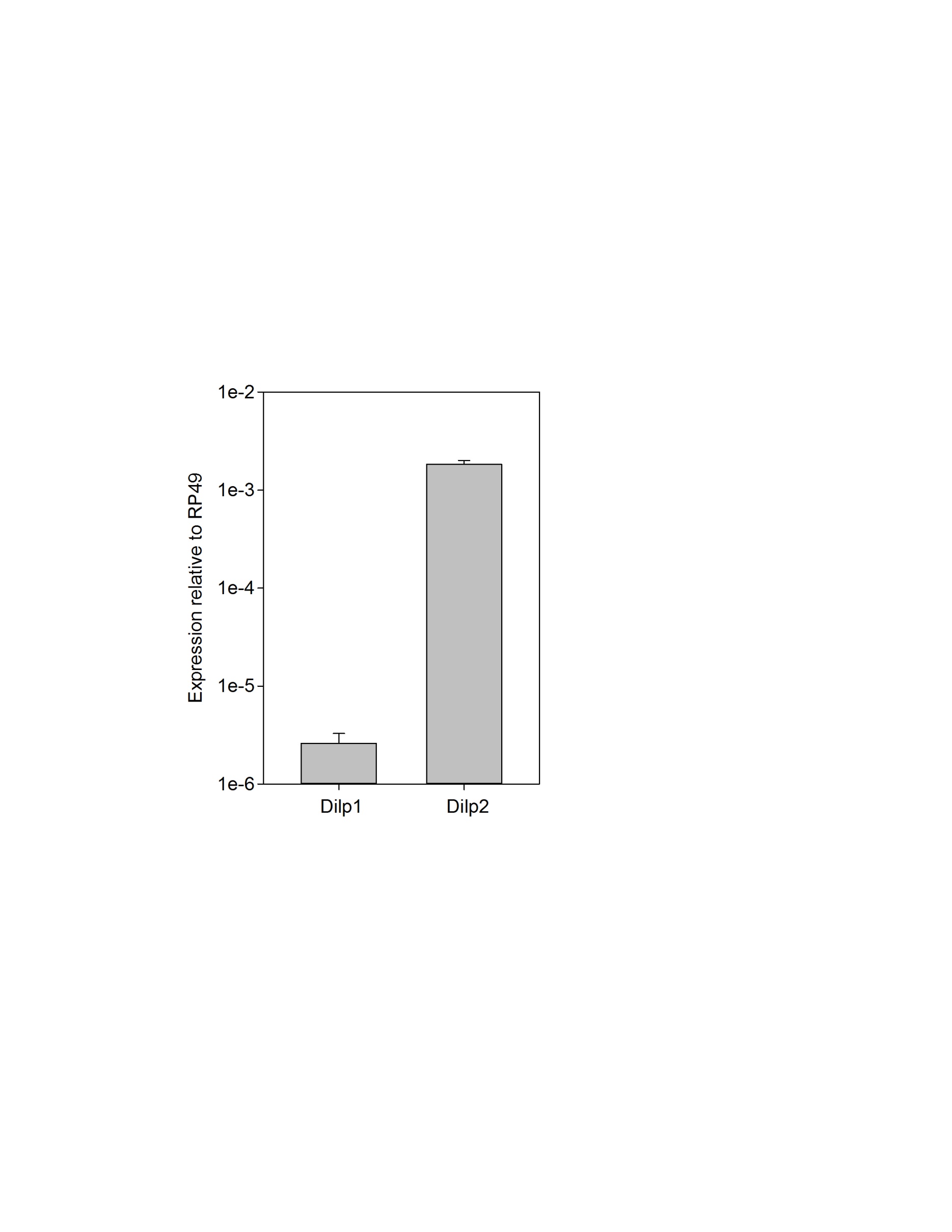

Supplement: S1 Fig — (DOCX) [file pone.0155628.s001.docx]
