# Supplementary material for: Nutritional Geometric Profiles of Insulin/IGF Expression in Drosophila melanogaster
Source: PLoS One. 2016 May 12;11(5):e0155628. doi: 10.1371/journal.pone.0155628 (PMC4865203; doi:10.1371/journal.pone.0155628)

S2 Fig. Upd2 expression along the geometric topography is hidden by extremely high expression at one outlier diet (Grubbs/ESD test p<0.05).


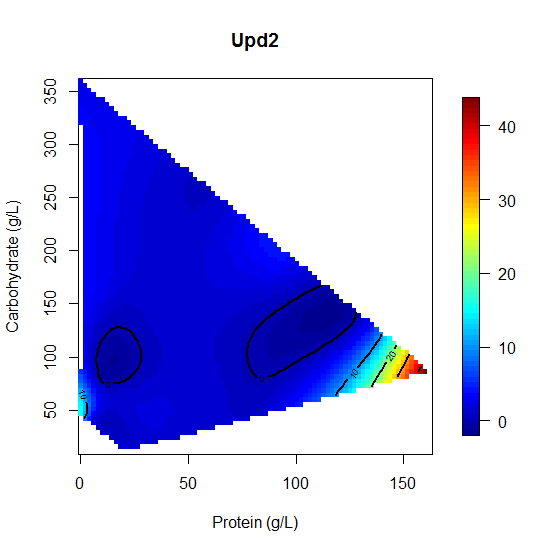

Supplement: S2 Fig — (DOCX) [file pone.0155628.s002.docx]
