# Supplementary material for: Nutritional Geometric Profiles of Insulin/IGF Expression in Drosophila melanogaster
Source: PLoS One. 2016 May 12;11(5):e0155628. doi: 10.1371/journal.pone.0155628 (PMC4865203; doi:10.1371/journal.pone.0155628)
Supplement: S1 Table — (PDF) [file pone.0155628.s003.pdf]

S1 Table. Relative gene expression values are the average of three technical replicates and are standardized to RP49 expression.

	Dilp1	Dilp2	Dilp3	Dilp4	Dilp5	Dilp6	Dilp7	Dilp8	Upd2	4eBP	InR
Diet 1	7.83E-05	3.61E-03	1.53E-03	2.37E-05	4.87E-04	1.87E-03	2.59E-04	1.43E-02	7.34E-05	1.95E+01	5.26E-03
Diet 9	5.50E-05	5.00E-03	3.03E-03	4.60E-04	1.33E-03	4.55E-03	5.62E-04	1.57E-02	9.93E-06	2.00E+01	1.43E-03
Diet 2	1.11E-04	5.21E-03	1.48E-03	3.31E-05	6.43E-04	5.37E-03	3.49E-04	4.18E-03	3.31E-05	2.41E+01	2.94E-03
Diet 10	2.75E-05	3.89E-03	3.10E-03	6.79E-05	1.35E-03	2.92E-03	1.38E-03	3.93E-02	1.54E-06	4.42E+01	8.19E-04
Diet 3	4.90E-05	2.86E-03	7.72E-04	4.44E-05	4.26E-04	2.29E-03	5.49E-04	1.63E-02	7.37E-06	2.82E+01	1.82E-03
Diet 11	2.00E-05	2.30E-03	1.40E-03	1.65E-04	8.32E-04	3.42E-03	6.18E-04	5.33E-03	6.83E-06	3.40E+01	1.26E-03
Diet 4	1.57E-05	1.72E-03	4.33E-04	4.04E-05	2.78E-04	1.19E-03	5.20E-04	2.13E-02	5.08E-06	4.13E+01	9.87E-04
Diet 12	3.52E-05	1.93E-03	1.20E-03	1.39E-04	7.93E-04	1.96E-03	5.37E-04	4.25E-03	7.13E-06	9.33E+01	2.62E-03
Diet 5	3.97E-05	2.71E-03	1.07E-03	2.59E-05	1.11E-03	2.83E-03	1.05E-03	2.39E-02	3.66E-06	4.60E+01	3.38E-03
Diet 13	2.30E-05	1.92E-03	1.39E-03	1.56E-05	1.48E-03	3.85E-03	1.02E-03	3.85E-02	8.02E-06	1.82E-01	2.10E-03
Diet 6	2.05E-05	1.63E-03	8.95E-04	1.60E-05	7.29E-04	2.29E-03	9.13E-04	3.09E-02	6.24E-06	4.46E+01	2.59E-03
Diet 14	3.00E-05	2.47E-03	1.13E-03	4.21E-05	1.12E-03	1.93E-03	1.39E-03	5.04E-02	5.64E-06	4.10E-01	2.77E-03
Diet 7	2.95E-05	1.68E-03	3.77E-04	5.64E-05	6.68E-04	2.37E-03	7.23E-04	2.21E-02	4.62E-06	3.97E+01	7.36E-03
Diet 15	8.34E-05	4.57E-03	1.34E-03	8.70E-05	5.02E-04	3.37E-03	6.66E-04	5.09E-02	1.85E-05	4.55E-01	1.97E-03
Diet 8	7.81E-05	4.53E-03	1.68E-03	7.95E-05	5.93E-04	3.39E-03	5.01E-04	3.38E-02	2.29E-05	2.42E+01	3.54E-03
Diet 16	5.74E-05	4.71E-03	1.63E-03	4.10E-05	9.54E-04	2.67E-03	5.67E-04	3.82E-02	1.22E-05	4.59E-01	2.00E-03
Diet 17	5.72E-05	2.56E-03	8.31E-04	1.72E-04	4.78E-04	2.39E-03	5.18E-04	2.95E-02	4.72E-06	5.35E-01	1.78E-03
Diet 23	6.01E-05	3.32E-03	1.70E-03	4.83E-04	8.36E-04	2.79E-03	5.73E-04	2.47E-02	1.15E-05	3.30E-01	2.00E-03
Diet 18	3.16E-05	3.01E-03	1.81E-03	4.85E-05	1.31E-03	2.52E-03	1.10E-03	4.72E-02	3.92E-06	2.44E-01	1.01E-03
Diet 24	5.40E-05	3.70E-03	2.25E-03	5.50E-04	9.94E-04	2.41E-03	1.77E-03	6.24E-02	8.28E-06	3.62E-01	2.02E-03
Diet 19	4.43E-05	2.47E-03	1.53E-03	1.31E-04	1.53E-03	2.91E-03	1.49E-03	7.04E-02	5.88E-06	3.41E-01	3.14E-03
Diet 25	2.66E-05	2.62E-03	1.03E-03	1.00E-04	7.05E-04	1.81E-03	3.81E-04	2.12E-02	4.35E-06	6.09E-01	1.42E-03
Diet 20	3.06E-05	2.03E-03	8.91E-04	1.98E-05	8.73E-04	2.37E-03	6.67E-04	3.86E-02	4.49E-06	3.37E-01	1.90E-03
Diet 26	3.48E-05	3.01E-03	1.71E-03	6.54E-05	2.01E-03	4.15E-03	1.26E-03	5.25E-02	2.01E-05	5.71E-01	3.48E-03
Diet 21	2.80E-05	1.46E-03	5.72E-04	7.34E-05	4.49E-04	2.44E-03	8.85E-04	4.10E-02	5.22E-06	3.08E-01	1.72E-03
Diet 27	6.63E-05	2.80E-03	1.39E-03	1.51E-04	7.93E-04	3.49E-03	1.17E-03	5.65E-02	1.69E-05	5.24E-01	3.31E-03
Diet 22	4.87E-05	3.79E-03	1.98E-03	6.33E-05	8.47E-04	2.79E-03	1.21E-03	7.29E-02	1.11E-05	3.53E-01	1.58E-03
Diet 28	1.20E-04	5.84E-03	2.66E-03	3.58E-05	9.78E-04	2.20E-03	5.68E-04	8.48E-03	2.10E-04	8.65E-01	8.61E-03
