# Supplementary material for: Nutritional Geometric Profiles of Insulin/IGF Expression in Drosophila melanogaster
Source: PLoS One. 2016 May 12;11(5):e0155628. doi: 10.1371/journal.pone.0155628 (PMC4865203; doi:10.1371/journal.pone.0155628)
Supplement: S2 Table — (DOCX) [file pone.0155628.s004.docx]

S2 Table. Multivariate multiple regression MANOVA table

| (Dilp1, Dilp2, Dilp3, Dilp4, Dilp5, Dilp6, Dilp7, Dilp8, Upd2, X4eBP, InR) ~ Ratio*Calorie | | | | | | | |
| --- | --- | --- | --- | --- | --- | --- | --- |
| Type II MANOVA Tests: Pillai test statistic | | | | | | | |
|  | Df | test stat | approx F | num Df | den Df | Pr(>F) |  |
| Ratio | 1 | 0.7062 | 3.0588 | 11 | 14 | 0.02616 |  |
| Calorie | 1 | 0.6478 | 2.3412 | 11 | 14 | 0.06818 |  |
| Ratio*Ratio | 1 | 0.70444 | 3.4667 | 11 | 16 | 0.01213 |  |
| Calorie*Calorie | 1 | 0.63711 | 2.5537 | 11 | 16 | 0.04326 |  |
| Ratio*Calorie | 1 | 0.7478 | 3.7745 | 11 | 14 | 0.01107 |  |
