# Supplementary material for: Nutritional Geometric Profiles of Insulin/IGF Expression in Drosophila melanogaster
Source: PLoS One. 2016 May 12;11(5):e0155628. doi: 10.1371/journal.pone.0155628 (PMC4865203; doi:10.1371/journal.pone.0155628)
Supplement: S3 Table — (DOCX) [file pone.0155628.s005.docx]

S3 Table. q-RT-PCR primer sequences

| Gene | Reference | Sequences |
| --- | --- | --- |
| dilp1 | Lee et al, 2009 | F 5’ GGGGCAGGATACTCTTTTAG 3’ |
|  |  | R 5’ TCGGTAGACAGTAGATGGCT 3’ |
| dilp2 | Lee et al, 2009 | F 5’ GTATGGTGTGCGAGGAGTAT 3’ |
|  |  | R 5’ TGAGTACACCCCCAAGATAG 3’ |
| dilp3 | Lee et al, 2009 | F 5’ AAGCTCTGTGTGTATGGCTT 3’ |
|  |  | R 5’ AGCACAATATCTCAGCACCT 3’ |
| dilp4 | (this study) | F 5’ GGCACCTGGATGTGATTTGTG 3’ |
|  |  | R 5’ GCTTTAACCTTCCCGTTTCC 3’ |
| dilp5 | Lee et al, 2009 | F 5’ AGTTCTCCTGTTCCTGATCC 3’ |
|  |  | R 5’ CAGTGAGTTCATGTGGTGAG 3’ |
| dilp6 | Okamoto et al, 2009 | F 5’ TGCTAGTCCTGGCCACCTTGTTCG 3’ |
|  |  | R 5’ GGAAATACATCGCCAAGGGCCACC 3’ |
| dilp7 | Okamoto et al, 2009 | F 5’ GAGCTGTACTCCTGTTCGTCCTGC 3’ |
|  |  | R 5’ TCCAAGCCTCATCATTGCCCGTCC 3’ |
| dilp8 | (this study) | F 5’ CTCAGCGAACTGGACATCTTT 3’ |
|  |  | R 5’ CACTGGTTTAGACAGCAGTAGG 3’ |
| Upd2 | (this study) | F 5’ AGCTAAAGACTTGGTACCGCCACA 3’ |
|  |  | R 5’ TCTTCTGCTGATCCTTGCGGAACT 3’ |
| 4eBP | Bai et al, 2013 | F 5’ CCATGATCACCAGGAAGGTTGTCA 3’ |
|  |  | R 5’ AGCCCGCTCGTAGATAAGTTTGGT 3’ |
| InR | (this study) | F 5’ ACCTATTTAACCACAAGCGA 3’ |
|  |  | R 5’ CTCGATAGTTCCAAGATTGC 3’ |
| RP49 | Bai et al, 2013 | F 5’ AAGAAGCGCACCAAGCACTTCATC 3’ |
|  |  | R 5’ TCTGTTGTCGATACCCTTGGGCTT 3’ |
